# Supplementary material for: Isolation and Characterization of the Primary Marmoset (Callithrix jacchus) Retinal Pigment Epithelial Cells
Source: Cells. 2023 Jun 16;12(12):1644. doi: 10.3390/cells12121644 (PMC10296975; doi:10.3390/cells12121644)
Supplement: Supplementary file 1 [file cells-12-01644-s001.zip › cells-2414796-supplementary.pdf]

Supplementary Materials:

Table S1. List of used antibodies.

Antibody	Company	Catalog No.	Dilution
ZO-1 antibody, Alexa FluorTM 594	Invitrogen	339194	1:100(IHC)
Phalloidin antibody, Alex Fluor™ 488	Invitrogen	A12379	1:100(IHC)
Antibody rabbit anti-occludin	Cell signaling technology	91131	1:100(IHC)
Anti-rabbit IgG secondary antibody, Alexa Fluor™ 488	Invitrogen	A11008	1:200(IHC)
Antibody rabbit anti-ZO-1	Cell signaling technology	8193	1:1000(WB)
Antibody rabbit anti-E-Cadherin	Cell signaling technology	3195	1:1000(WB)
Antibody mouse anti-Ezrin	ThermoFisher Scientific	3C12	1:1000(WB)
Antibody mouse anti-RPE65	Novus Biologicals	401.8B11.3D9	1:500(WB)
Antibody mouse anti-F-actin	Abcam	AB205	1:500(WB)
Antibody rabbit anti-GAPDH	Cell signaling technology	5174	1:1000(WB)
Antibody mouse anti-p53	Santa Cruz Biotechnology	sc-126	1:200(WB)
Antibody mouse anti-p21	Santa Cruz Biotechnology	sc-6246	1:200(WB)
Antibody rabbit anti-CDK2	Cell signaling technology	2546	1:1000(WB)
Antibody rabbit anti-CDK4	Cell signaling technology	12790	1:1000(WB)
Antibody mouse anti-CDK6	Cell signaling technology	3136	1:1000(WB)
Antibody mouse anti- Cyclin A2	Cell signaling technology	4656	1:1000(WB)
Antibody mouse anti- Cyclin D1	Cell signaling technology	2926	1:1000(WB)
Antibody rabbit anti- Cyclin E2	Cell signaling technology	4132	1:1000(WB)
Anti-rabbit IgG	Cell signaling technology	7074	1:2000(WB)
Anti-mouse IgG	Cell signaling technology	7076	1:2000(WB)
